# Molecular characterization and functional analysis of DIGIRR from golden pompano (*Trachinotus ovatus*)

**DOI:** 10.3389/fimmu.2022.974310

**Published:** 2022-08-24

**Authors:** Yushuai Xie, Shuangshuang Gao, Yiwen Cao, Yuexin Ji, Qihuan Zhang, Youchuan Wei, Zhitao Qi

**Affiliations:** ^1^ College of Animal Science and Technology, Guangxi University, Nanning, China; ^2^ Jiangsu Key Laboratory of Biochemistry and Biotechnology of Marine Wetland, Yancheng Institute of Technology, Yancheng, China

**Keywords:** *Trachinotus ovatus*, DIGIRR, immune response, subcellular localization, co-IP

## Abstract

Mammalian single immunoglobulin (Ig) interleukin-1 receptor related molecule (SIGIRR), an important member of the Toll/interleukin-1 receptor (TIR) family, plays important balancing roles in the inflammatory responses. In the present study, the double Ig interleukin-1 receptor related molecule (DIGIRR), the homologous of SIGIRR, was characterized in golden pompano (*Trachinotus ovatus*) (termed as trDIGIRR). The full-length cDNA of trDIGIRR was 2,167 bp with an open reading frame (ORF) of 1,572 bp encoding 523 amino acids. The trDIGIRR contained several conserved domains including a signal peptide, two Ig domains, a transmembrane domain and a TIR domain, and shared high sequence identities with its teleost counterparts. Realtime qPCR analysis revealed that the trDIGIRR was distributed in all tissues examined, with high expressions in intestine, liver and head kidney. The expressions of trDIGIRR were induced by *Vibrio alginolyticus*, lipopolysaccharide (LPS) and polyinosinic-polycytidylic acid (poly I:C) challenge. Further analysis revealed that trDIGIRR was mainly located in the cytoplasm. In addition, the co-immunoprecipitation (co-IP) assay identified that trDIGIRR could interact with myeloid differentiation factor 88 (MyD88), but not interact with TIR domain containing adaptor protein inducing interferon-β (TRIF). Our results provide basis for studying the immune role of fish DIGIRR.

## Introduction

Toll-like receptors (TLRs) are important pattern recognition receptors (PRRs) involving in regulating inflammatory response of host (1, 2). Structurally, TLRs contain several conserved motifs, including the extracellular leucine-richrepeat (LRR) motifs, a transmembrane domain and an intracellular toll/interleukin-1 receptor (TIR) domain (3, 4). Once LRR motifs recognize and bind the pathogen-associated molecular patterns (PAMPs), the myeloiddifferentiationfactor 88 (MyD88) -dependent or TIR-domain-containing adaptor inducing interferon-β (TRIF) -dependent signaling pathway will be activated (5–8), leading to the secretion of cytokines, such as interleukin (IL)-1, IL-6, IL-8, tumor necrosis factor (TNF) α and interferon (IFN) γ, and thereby enhancing the inflammatory and immune responses (9–12). However, excessive immune response triggers an imbalance between anti-inflammatory and proinflammatory which in turn causes tissue damages (13, 14).

Single immunoglobulin (Ig) IL-1 receptor related molecule (SIGIRR), also named as TIR8, is an important member of TIR family, which contains an extracellular Ig domain that involves in recognizing ligands, a transmembrane domain and an intracellular TIR domain that functions in the transduction of downstream signals (15, 16). Mammalian SIGIRR is the suppressor of TLRs signaling (17–19). When the inflammation is over-reacted, SIGIRR inhibits the homodimerization of TRIF-related adaptor molecule (TRAM) and the interaction between TRIF and TRAM (20–22). Also, SIGIRR can interact with MyD88 or TRAF6 by its TIR domain to interfere the recruitment of adaptors of TLRs signaling (23). In addition, SIGIRR-deficient mice had an enhanced response to lipopolysaccharide (LPS) and IL-1 stimulation, and showed an increasing number of neutrophils and macrophages in lungs (18, 24). *In vitro* experiments revealed that SIGIRR-deficient tubular epithelial cells had an increasing production of cytokines or chemokines under the stimulation of LPS or heat-killed *Escherichia coli* (25). These studies indicate an anti-inflammatory role of SIGIRR.

Two types of SIGIRRs were identified in teleosts. The one type of SIGIRR, similar to human SIGIRR, only contains a single Ig domain and is mainly located on the cell membrane (26). The other type of SIGIRR contains two Ig domains, designated as DIGIRR, and is mainly distributed in the cytoplasm (27). Interestingly, no fish contains both types of SIGIRRs. So far, DIGIRR has been reported in Japanese pufferfish (*Takfugu rubripes*) (28), miiuy croaker (*Miichthys miiuy*) (27) and Chinese sturgeon (*Acipenser sinensis*) (29), whereas SIGIRR was only identified in zebrafish (*Danio rerio*) (26) and grass carp (*Ctenopharyngodon idella*) (30).

Golden pompano (*Trachinotus ovatus*), an important marine aquaculture fish, is widely distributed in tropical and subtropical oceans (31). The annual production of golden pompano has reached approximate 120 thousand tons in South China (32). However, the occurrence of diseases, especially the pathogenic diseases, increased yearly which caused huge economic losses to the industry of golden pompano (33). Studying the functions of DIGIRR may contribute to preventing the diseases of golden pompano as most diseases are accompanied by inflammatory response. In the present study, the DIGIRR was cloned and characterized from golden pompano (trDIGIRR), and its expressions following LPS, polyI:C and bacterial infection were investigated. Further, the interaction between trDIGIRR and other adaptors of TLRs signaling pathway were verified by co-IP assay. Our study provides a solid basis for understanding the functions of DIGIRR in golden pompano.

## Materials and methods

### Fish and tissues collection

Healthy golden pompanos (250 ± 15.2 g) were kept in the running seawater at 26 ± 2°C in our laboratory for at least 2 weeks before experiments. The fishes were randomly divided into four groups (30 fishes per group): LPS challenge group, poly I:C challenge group, *Vibrio alginolyticus* challenge group and control group, among which the fishes were intraperitoneally (i.p) injected with LPS (100 μg per 100 g fish, Sigma, USA), poly I:C (100 μg per 100 g fish, Sigma, USA), 5×10^7^ CFU live *V. alginolyticus* (100 μL per 100 g fish, TSTO, China) and same amount of PBS solution, respectively. The head kidney (HK), spleen, liver, intestine and gill were collected from five fishes in each group at 0 h, 6 h, 12 h, 24 h, and 48 h post-injection (hpi). In addition, tissues including spleen, HK, skin, muscle, gill, heart, liver, brain and intestine were respectively collected from five healthy fishes. All tissues were immediately frozen in liquid nitrogen and stored at −80°C until use.

### RNA extraction and cDNA synthesis

Total RNA was isolated using Trizol reagent (TaKaRa, Japan) according to the manufacturer’s instructions, and the concentration and quality of total RNA were assessed by 1.0% agarose gel electrophoresis and spectrophotometry (Eppendorf, Germany). The total RNA was transcribed into the first-strand cDNA by using First Strand cDNA Synthesis Kit (Thermo Fisher Scientific, USA) following the manufacturer’s protocol.

### Cloning of trDIGIRR

Degenerate primers were designed to amplify the partial cDNA sequence of trDIGIRR based on the DIGIRR sequences of greater amberjack (*Seriola dumerili)* (XM_022755944.1), silver sea perch (*Lates calcarifer*) (XM_018666410) and large yellow croaker (*Larimichthys crocea*) (XM_019253995.1). The first round of PCR amplification was performed in 25 μL reaction mixtures containing 12.5 μL of 2 × *EasyTaq* ^®^ PCR SuperMix (TaKaRa, Japan), 1 μL of first strand cDNA, 1 μL of each primer and 9.5 μL of RNase-free water. The PCR program was: an initial denaturing cycle at 94°C for 4 min, 5 cycles of 94°C for 30 s, 67°C for 30 s, 72°C for 90 s, 5 cycles of 94°C for 30 s, 65°C for 30 s, 72°C for 90 s, followed by 20 cycles of 94°C for 30 s, 63°C for 30 s, 72°C for 90 s and a final elongation at 72°C for 10 min. The second round PCR reaction was same as that of the first round PCR. The products of three times PCR were confirmed by sequencing on an automatic DNA sequencer (Gigascience, China).

The full cDNA length of trDIGIRR was obtained by RACE PCR usingthe SMART RACE cDNA Amplification Kit (TaKaRa, Japan) by following the manufacturer’s instruction.The first round of 5’-end RACE PCR was performed using universal primers (UPM) and DIGIRR-5R1, and the PCR program was: denaturation at 94°C for 4 min, 5 cycles of 94°C for 30 s, 68°C for 30 s, 72°C for 50 s, 5 cycles of 94°C for 30 s, 66°C for 30 s, 72°C for 50 s, followed by 20 cycles of 94°C for 30 s, 64°C for 30 s, 72°C for 50 s and a 10 min of extension at 72°C. The first round of 3’-end RACE PCR was performed using UPM and DIGIRR-3F1 primers, and used the same PCR program as 5’-end RACE PCR except that the annealing temperature was 66°C. The primers of the second round of 5′- and 3′-end RACE PCR were the nested universal primer (NUP)/DIGIRR-5R1 and NUP/DIGIRR-3F1, respectively, and the PCR programs were same as that of first round RACE PCR. The PCR products were analyzed by electrophoresis in 1.0% agarose gel and subsequently purified using an agarose gel DNA purification kit (Sangon, China). Then, the purified products were ligated into the pMD18-T vector (TaKaRa, Japan) and sequenced on an automatic DNA sequencer (Gigascience, China). The full-length cDNA of trDIGIRR was assembled by the sequencing results of four clones using Seqman software. All the primers used for gene cloning were listed in **Table 1**.

**Table 1:** Primers in this study.

Primer Name	Primer sequence (5’-3’)	Use
DIG-F1	TGTGGATGAGAGCAGGTTTAAGGA	trDIGIRR Partial sequence
DIG-R1	GGCAGTAGAAGTCTGAGCGGGC	
DIG-F2	TGCGTCTCATAGTTAAAGAGTCCCA	
DIG-R2	GTGATCCCAAGTCCGACACGTC	
DIG-5R1	GCGTGGTGTCACATTGCTCCTC	trDIGIRR 5’UTR
DIG-5R2	CCACAGTCCGTTCAGGTCTCCA	
DIG-3F1	ATGCTGACCCTTGAACCCGACT	trDIGIRR 3’UTR
DIG-3F2	GCTCAGACACTGACCCTGCTGG	
UPM-L	CTAATACGACTCACTATAGGGCAAGCAGTGGTATCAACGCAGAGT	RACE universal primers
UPM-S	CTAATACGACTCACTATAGGGC	
NUP	AAGCAGTGGTATCAACGCAGAGT	
DIG-qF	CTTACCTGGCGGAGAACCTT	trDIGIRR expression
DIG-qR	ACGGGCTGCTGACATAACTC	
β-actin-F	GCTACGTCGCCCTGGACTTC	Gene expression
β-actin-R	CTCATGGATTCCGCAGGACTC	
DIG-ORF-F	CGGAGGAAGAGGAGACTTGCGG	trDIGIRR ORF
DIG-ORF-R	GAGATCAGGTGAGGGGAATGGG	
DIG-NI-F	CCCAAGCTTATGGCTGCGATTGTAGTC	pEGFP-N1-trDIGIRR construction
DIG-NI-R	GGGGTACCGTAATGTCCTCAGTGACCAG	
DIG-3.1-F	GGGGTACCATGGCTGCGATTGTAGTC	pcDNA3.1-trDIGIRR-His construction
DIG-3.1-R	ATAGTTTAGCGGCCGCTTAATGATGATGATGATGGTGAATGTCCTCAGTGACCAGGC	
MyD88-3.1-F	CCGGAATTCATGGCTTGTGCTGAGACAGATGTT	pcDNA3.1-trMyD88-Flag construction
MyD88-3.1-R	CCGCTCGAGTTACTTATCGTCGTCATCCTTGTAATCCGGCAGTGAAAGAGCTTT	
TRIF-3.1-F	CCCAAGCTTATGAGTCACGAGGGACAAGA	pcDNA3.1-trTRIF-Flag construction
TRIF-3.1-R	CCGGAATTCTTACTTATCGTCGTCATCCTTGTAATCTTGCTCCTCCTCTGAGGAAC	

The UPM primer used in RACE-PCR were mixed with UPM-S primer and UPM-L primer at the ratio of 5:1.

### Sequence analysis

The assembled sequence of trDIGIRR was annotated using the BLASTX program (http://blast.ncbi.nlm.nih.gov/Blast.cgi), and the corresponding amino acids, theoretical molecular weight (MW) and isoelectric point (pI) were deduced using The ExPASy website (http://web.expasy.org/translate/). The signal peptide was predicted by SignalP 5.0 software (http://www.cbs.dtu.dk/services/SignalP/). The secondary and three-dimensional structures of DIGIRR or SIGIRR were predicted using Protean software, Alpha fold 2 software and SWISS-MODEL software (https://swissmodel.expasy.org/). Sequence alignments were done by the MegAlign software in the DNAstar 7 software package (http://www.dnastar.com/). Multiple sequence alignment of proteins was performed with Clustal O software (http://www.clustal.org/omega/) and decorated using GeneDoc software (http://www.nrbsc.org/gfx/genedoc/index.html). The protein domains were predicted using the Simple Modular Architecture Research Tool (SMART) (http://smart.embl-heidelberg.de/). Phylogenic tree was constructed by the neighbor-joining method using MEGA 7.0 software with the bootstrap settingas 10,000.

### qPCR

The expressions of trDIGIRR were determined on the LightCycler480 (Roche, Germany) qPCR system. The qPCR reaction was 20 μL containing 10 μL of 2×TransStart^®^ Top Green qPCR SuperMix (TransGen Biotech, China), 0.4 μL of each primer (0.2 μM), 1μL of cDNA template, 8.2 μL of RNase-free water. The qPCR reaction program was 94°C for 2 min; 40 cycles of 94°C for 30 s, 62°C for 30 s, 72°C for 20 s; and 37°C for 2 min. All qPCR reactions were performed in triplicate. The qPCR data was analyzed using Roche LightCycler^®^96 PCR system toobtain Ct value of trDIGIRR and β-actin (internal reference). The relative expressions of trDIGIRR in normal tissues were normalized to that of β-actin. The transcript changes of trDIGIRR gene in different tissues following LPS, poly I:C, *V. alginolyticus* and PBS infection were calculated using the 2^−△△CT^ method (34). All primers used for qPCR were listed in **Table 1**.

### Plasmids construction

The open reading frame (ORF) of trDIGIRR was amplified by PCR with specific primers containing *Kpn* I and *Not* I cleavage sites. The ORF of trMyD88 was amplified using the specific primers containing *EcoR* I and *Xho* I cleavage sites, while the ORF of trTRIF was amplified using the specific primers containing *Hind* III and *EcoR* I cleavage sites. The PCR products were ligated into pcDNA3.1 vector (Invitrogen, USA) and digested using corresponding enzymes (TaKaRa, Japan). After the plasmids were confirmed by sequencing, the plasmids were extracted with endotoxin-freeplasmid DNA Miniprep Kit (TransGen Biotech, China) and designated as pcDNA3.1-trDIGIRR-His, pcDNA3.1-trMyD88-Flag and pcDNA3.1-trTRIF-Flag, respectively. In addition, Subcellular localization plasmid was conducted by fusing trDIGIRR with pEGFP-N1 (Clontech, USA) and named as pEGFP-N1-trDIGIRR. All the primers used for plasmids constructing were listed in **Table 1**.

### Cell culture and transfection

HEK-293T cells (Thermo Fisher Scientific, USA) were cultured in Dulbecco’s Modified Eagle Medium (DMEM, Invitrogen, USA) supplemented with 10% Fetal Bovine Serum (FBS, Invitrogen, USA), penicillin (10 U/mL), and streptomycin (100 μg/L) at 37°C in 5% CO_2_. Cells were sub-cultured and seeded onto coverslips in 24-well plates before transfection. After twenty-four hours, cells culturing in Opti-MEM (Invitrogen, USA) medium without serum, were transfected with 500 ng expression plasmid or an empty vector using Lipofectamine 2000 reagent (Invitrogen, USA) according to the manufacturer’s instructions.

### Subcellular localization of trDIGIRR

HEK-293T cells were sub-cultured into 24-well plates and cultured until reaching about 80% confluence. The cells were transfected with the recombinant pEGFP-N1-trDIGIRR and pEGFP-N1 expression plasmid, respectively. At 48 h post transfection, the cells were washed three times with PBS and fixed with 4% paraformaldehyde (Beyotime, China) at room temperature for 20 min. Then, the cells were stained with DAPI (1 μg/mL, Solarbio, China) fluorescence and determined to observe the localization of trDIGIRR protein by fluorescence microscope TH4-200 (OLYMPUS, Japan).

### Coimmunoprecipitation and western blot analysis

HEK-293T cells were sub-cultured into two 6-well plates and cultured until reaching about 80% confluence. The cells were co-transfected with pcDNA3.1-trDIGIRR-His/pcDNA3.1-trMyD88-Flag or pcDNA3.1-trDIGIRR-His/pcDNA3.1-trTRIF-Flag, respectively. At 48 h post transfection, the cells were lysedfor 30 min on ice using 300 μL of NP-40 lysis buffer. The NP-40 lysis buffer was prepared with NP-40 (Beyotime, China) and PMSF (Beyotime, China) in a ratio of 1:100. The whole cell lysates were collected into a pre-cooled 1.5 mL sterilized tube. After centrifuged at 4°C (14000 g for 15 min), the supernatant was transferred to a new tube. The total protein (1.2 mL) was divided into three groups (Group A, B and C). For each group, 40 μL of protein A+G beads (50% in PBS, Beyotime, China) were added into 400 μL total protein respectively, and then the specific hybrid protein was removed by incubation on a rotator for 30 min at 4°C. Further, His-tag antibody (HRP Conjugated, Abmart, China) was added into the total protein of Group A, Flag-tag antibody (HRP Conjugated, Abmart, China) was added into Group B, while Group C was the total protein without Co-IP treatment (input group). After incubation on a rotator at 4°C overnight, 40 μL of protein A+G beads were added into each group and incubated overnight on a rotator at 4°C. The complexes were collected by centrifuging at 4°C (2000 g for 3 min) and washed three times with pre-cooled PBS. Then, pre-cooled PMSF, pre-cooled NP-40 and 5 × loading buffer were added into the mixtures and boiled for 10 min to separate the proteins, antibody and beads.

The protein samples were isolated by 12% acrylamide SDS-PAGE and transferred onto polyvinylidene fluoride (PVDF) membranes (Solarbio, China) using wet-transfer process. After transfer, the membrane was blocked overnight in Tris Buffered Saline with Tween (TBST) containing 5% skim milk (Solarbio, China) at 4°C and then incubated overnight with diluted primary antibody (HRP- Conjugated) at 4°C. Finally, signals were visualized using BeyoECL Plus kit (Beyotime, China) according to the manufacturer’s protocol.

### Statistical analysis

The qPCR data were calculated in GraphPad Prism 7.0 software and expressed as means ± standard error (SE). Statistical significance was analyzed using one-way of variance (ANOVA) method with SPSS 25.0 software. *P* value <0.05 was considered significant and *P* value < 0.01 was considered extremely significant.

## Results

### Sequence characteristics of trDIGIRR

**Figure 1:**
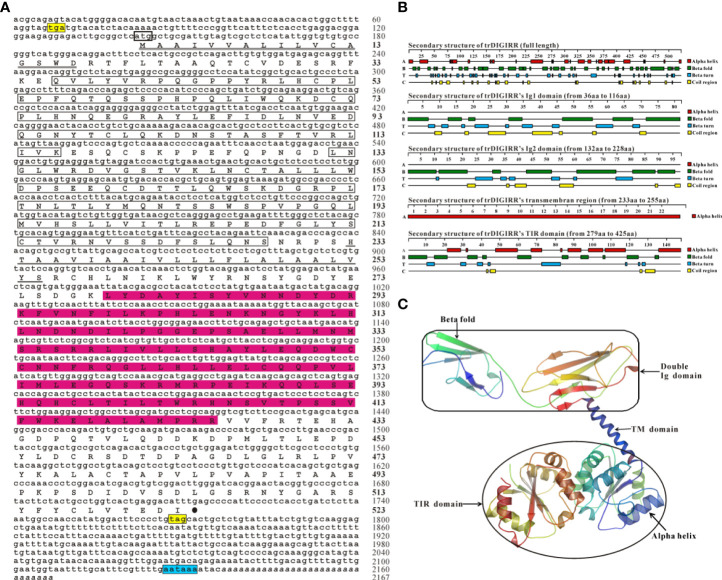
The nucleotide and deduced amino acid sequence **(A)**, secondary structure **(B)** and 3-D structure **(C)** of trDIGIRR. **(A)** The start codon was marked with a thick square, the stop codon was labeled with a black solid circle, the signal peptide was labeled with a double horizontal line, the Ig domain was marked with a square, the transmembrane region was marked with a horizontal line, the TIR domain was marked with a red background, and poly-A tail was italicized. Two in-frame stop condons were marked in yellow and the the polyadenylation signal (aataaa) upstream of the poly **(A)** tail was marked in blue. **(B)** The secondary structure of trDIGIRR was predicted using Protean software. **(C)** The 3-D structure of trDIGIRR was constructed using the SWISS-MODEL software.

**Figure 2:**
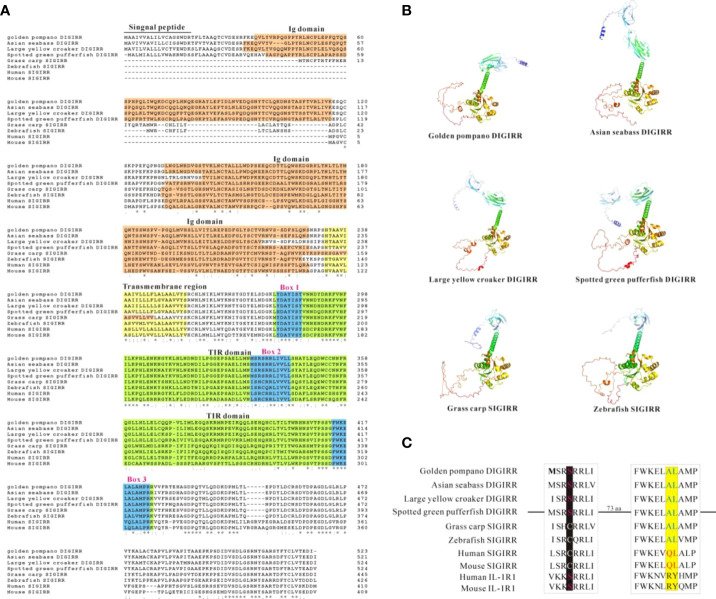
Comparison of DIGIRR, SIGIRR predicted amino acid sequence **(A)**, 3-D structure **(B)** and TIR domain key amino acid **(C)** in different vertebrates. The underlined part was the signal peptide and the red shaded part was the Ig domain. The yellow shaded part was transmembrane region. The green shaded part was TIR domain and the blue box (1-3) was the Box motif in the TIR domain. The 3-D structure was performed using Alpha fold 2 software. The GenBank accession numbers of each specie sequences were summarized in **Table 2**.

The full-length cDNA of trDIGIRR (GenBank accession number: MW239681) was 2,167 bp, including a 141 bp of 5’-untranslated region (UTR), a 454 bp of 3’-UTR with poly A tail and a 1,572 bp of ORF encoding 523 amino acids (aa) (**Figure 1A**). Two in-frame stop condons were respectively found in the 5’-UTR and 3’-UTR. In addition, the polyadenylation signal (aataaa) upstream of the poly (A) tail and poly (A) tail were found in the 3’-UTR of DIGRIRR. These results indicated the complete cDNA sequence of the gene had been obtained. The trDIGIRR contained a signal peptide at its N-terminal (**Figure 1A**), and its predicted molecular weight and the theoretical pI were 59.38 kDa and 5.68, respectively. Protein domain prediction showed that trDIGIRR possessed the typical structural characteristics of IL-1R family, including two extracellular Ig domains (Ig1: from 36 aa to 116 aa; Ig2: from 132 aa to 228 aa), a transmembrane region (from 233 aa to 255aa), and an intracellular conserved TIR domain (from 279 aa to 425 aa) (**Figure 1A**). Further, the secondary structure analysis revealed that the extracellular Ig domains of trDIGIRR was consisted of 15 β-fold, 19 β-turn and 15-coil regions (**Figure 1B**), consistent with the 3-D structure of trDIGIRR (**Figure 1C**). Sequence alignment revealed that the TIR domain of trDIGIRR contained three Box motifs, Box1-3 (**Figure 2A**). Further analysis of the 3-D structures of fish DIGIRR or SIGIRR revealed that TIR domains of DIGIRR or SIGIRR were highly conserved, whilst the number of the Ig domains was variable. Only one Ig domain was found in fish SIGIRR, whilst two Ig domains were found in fish DIGIRR. The structures of the two Ig domains of fish DIGIRR were similar (**Figure 2B**). In addition, amino acid site alignment showed that the TIR domain of trDIGIRR has one mutation (Arg-Leu replaced Arg-Tyr) in the two amino acid residues (Ser and Arg-Tyr) (**Figure 2C**).

### Homology and phylogenetic analysis

**Figure 3:**
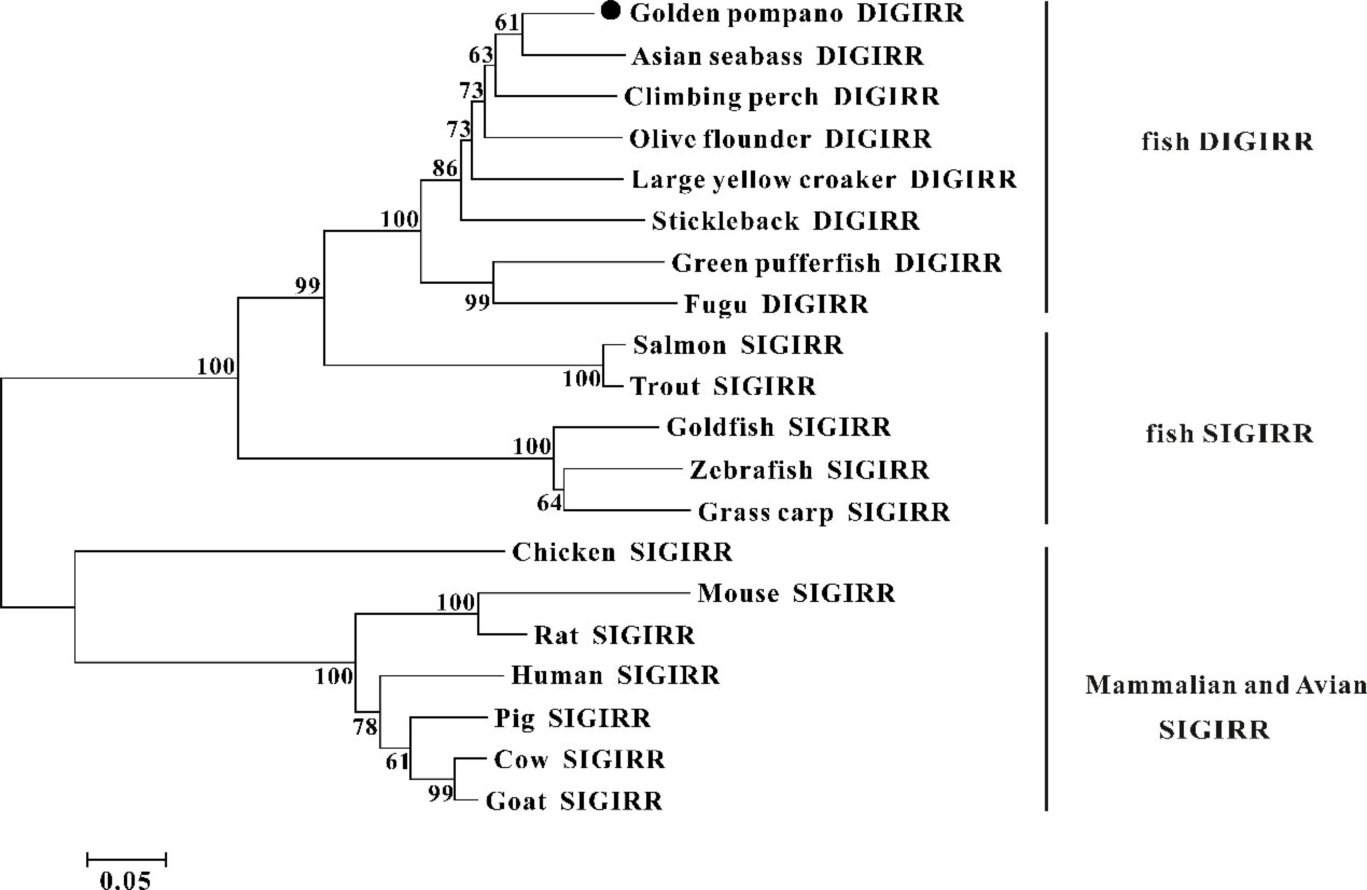
The phylogenetic tree of DIGIRR and SIGIRR genes in different species. The phylogenetic tree was constructed using the Neighbor-joining method (NJ) with 10000 bootstrapping replications by MEGA 7.0 software. The sequence information of all species was shown in **Table 2**.

The homology comparison showed that trDIGIRR had 68.5%-86.3% identities with fish DIGIRR, among which trDIGIRR shared the highest identity with *Lates calcarifer* DIGIRR (86.3%). In addition, trDIGIRR had 54.1%-68.5% identity with fish SIGIRR, but had low identity with mammalian SIGIRR (44.4%-51.2%) (**Table 2**). A phylogenetic tree was constructedto understand the evolutionary relationship between DIGIRR and SIGIRR in different vertebrates. The result showed that fish DIGIRR and SIGIRR were clustered together to form a separate cluster from these of mammals (**Figure 3**).

**Table 2:** Protein sequence identities between trDIGIRR and vertebrates’ DIGIRR/SIGIRR.

Gene Name	Species	Identities (%)	Genbank No.
DIGIRR	*Trachinotus ovatus*	***	MW239681
DIGIRR	*Lates calcarifer*	86.3	XP_018521926.1
DIGIRR	*Anabas testudineus*	83.0	XP_026220740.1
DIGIRR	*Paralichthys olivaceus*	81.7	XP_019934747.1
DIGIRR	*Larimichthys crocea*	79.9	XP_019109540.2
DIGIRR	*Gasterosteus aculeatus*	77.1	ACA51853.1
DIGIRR	*Takifugu rubripes*	73.2	NP_001266941.1
DIGIRR	*Tetraodon nigroviridis*	69.9	ABO15773.1
DIGIRR	*Salmo salar*	68.5	NP_001167309.1
SIGIRR	*Oncorhynchus mykiss*	68.5	XP_021434241.1
SIGIRR	*Carassius auratus*	59.2	XP_026095071.1
SIGIRR	*Danio rerio*	56.8	AEJ36290.1
SIGIRR	*Ctenopharyngodon idella*	54.1	QCX41148.1
SIGIRR	*Capra hircus*	51.2	XP_017898457.1
SIGIRR	*Bos taurus*	50.2	NP_001075912.1
SIGIRR	Sus scrofa	49.5	NP_001302618.1
SIGIRR	*Homo sapiens*	49.0	NP_001128526.1
SIGIRR	*Rattus norvegicus*	48.1	NP_001020058.1
SIGIRR	*Gallus gallus*	47.3	NP_001186471.1
SIGIRR	*Mus musculus*	44.4	AAF26200.1

***Means as 100%.

### Tissue distribution and subcellular localization of trDIGIRR

**Figure 4:**
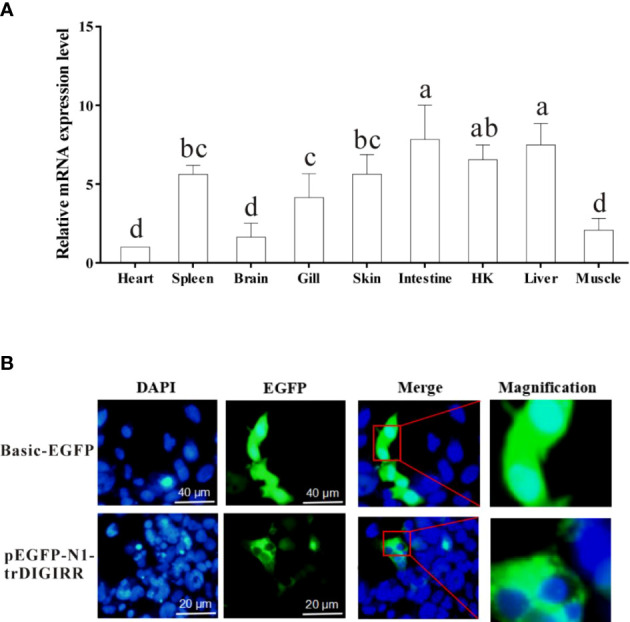
Relative mRNA expression level of DIGIRR in spleen, HK, skin, muscle, gill, heart, liver, brain and intestine of healthy golden pompano **(A)**, and the subcellular localization of trDIGIRR in HEK-293T cells **(B)**. A: Bars that do not share a letter represent a significant difference (*p* < 0.05).

The qPCR was used to detect the expression of trDIGIRR in nine tissues (spleen, HK, skin, muscle, gill, heart, liver, brain and intestine) of normal golden pompano. Results showed that trDIGIRR was constitutively distributed in all examined tissues (**Figure 4A**). Notably, trDIGIRR expression was relatively high in intestine, liver, HK and spleen, whereas was relatively low in heart and muscle.

To furtherclarify the biological function of trDIGIRR, EGFP-labeled trDIGIRR (pEGFP-N1-trDIGIRR) and pEGFP-N1 plasmids were transfected into HEK-293T cells, respectively. The results showed clearly that the trDIGIRR-expressing region (green) in HEK-293T cells was tightly surrounded the nucleus (**Figure 4B**), demonstrating that trDIGIRR may be a predominantly cytoplasmic protein.

### 3.4 Expression of trDIGIRR following LPS, poly I:C and *V. alginolyticus* challenge

**Figure 5:**
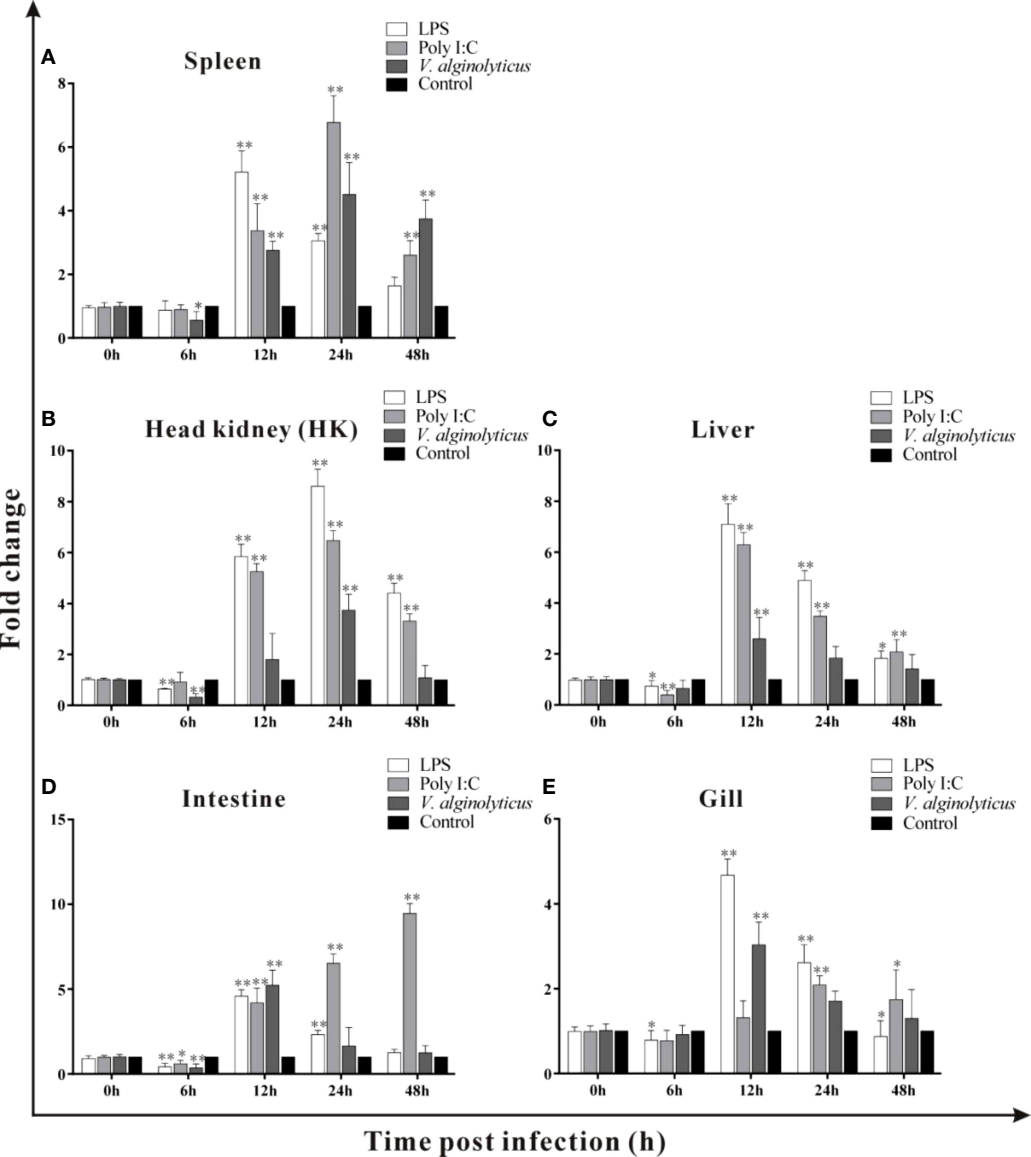
Expression patterns of trDIGIRR in spleen, head kidney (HK), liver, intestine and gill following the *Vibrio alginolyticus*, LPS and poly I:C challenge **(A–E)**. The relative expression of target gene in each tissue was normalized to that of β-actin. The expression changes of the two genes were analyzed using the 2^-△△CT^ method and expressed as fold change. Results were means ± SE.*P<0.05, **P<0.01.

Host-expressed pattern recognition receptors (PRRs) can recognize a series of pathogen-associated molecular patterns (PAMPs), such as LPS, ssRNA and dsRNA, and trigger various physiological responses including inflammation (35–38). LPS is the main components of gram-negative bacteria and polyI:C is mimic of dsRNA of virus (37). In the current study, the expressions of trDIGIRR following LPS, poly I:C (dsRNA) and *V. alginolyticus* were detected using qPCR (**Figures 5A–E**). Following LPS challenge, the expression of trDIGIRR in spleen was increased from 12 h to 24 h (**Figure 5A**). The transcript changes of trDIGIRR shared similar patterns following LPS challenge in HK and liver, which was decreased at 6 h, and increased from 12 h to 48 h (**Figures 5B, C**). The expressions of trDIGIRR in intestine (**Figure 5D**) and gill (**Figure 5E**) were also decreased at 6 h, but increased from 12 h to 24 post LPS challenge. Following polyI:C challenge, trDIGIRR was up-regulated in spleen (**Figure 5A**) and HK (**Figure 5B**) from 12 h to 48 h, and up-regulated in gill from 24 h to 48 h (**Figure 5E**). The expressions of trDIGIRR were similar in liver and intestine, which were down-regulated at 6 h and then up-regulated from 12 to 48 post polyI:C challenge (**Figures 5C, D**). Following *V. alginolyticus* infection, trDIGIRR was down-regulated at 6 h, and then up-regulated from 12 h to 48 h in spleen (**Figure 5A**). The expressions of trDIGIRR were only down-regulate in HK at 6 h (**Figure 5B**), but only up-regulated at 12 h in liver (**Figure 5C**) and gill (**Figure 5E**) following *V. alginolyticus* infection. In intestine, trDIGIRR was down-regulated at 6 h and then up-regulated at 12 h after *V. alginolyticus* infection (**Figure 5D**).

### The interaction between trDIGIRR and tyMyD88 or trTRIF

**Figure 6:**
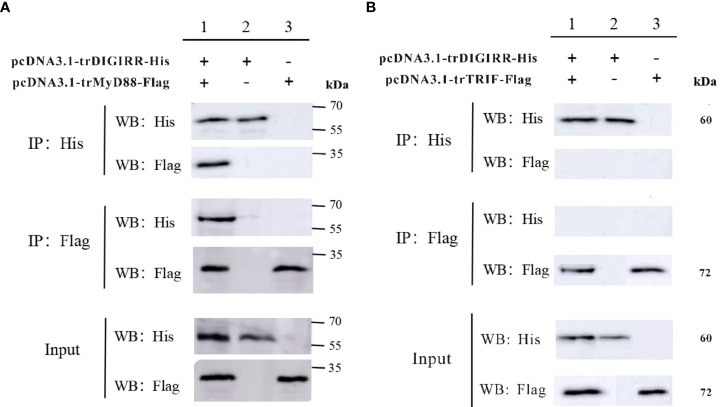
Co-immunoprecipitation analysis of the association between trDIGIRR with trMyD88 **(A)** or trTRIF **(B)**.

In order to explore the possible interaction between trDIGIRR and trMyD88 or trTRIF, two groups of co-IP experiments were performed, respectively. The results revealed pcDNA3.1-trDIGIRR-His and pcDNA3.1-trMyD88-Flag (or pcDNA3.1-trTRIF-Flag) proteins could be expressed in H293T cells (**Figure 6**). Further, in the co-IP of trDIGIRR and trMyD88, specific bands representing trDIGIRR (or trMyD88) were detected in the precipitated proteins by trMyD88 or trDIGIRR (**Figure 6A**), whereas in the co-IP of trDIGIRR and trTRIF, no specific bands were observed in the precipitate proteins by trTRIF or trDIGIRR (**Figure 6B**), which demonstrated that trDIGIRR interacted with trMyD88 but not with trTRIF.

## Discussion

TLRs play crucial roles in the activation of host innate and adaptive immunity (39). However, excessive TLRs activation can disrupt immune homeostasis, andmay be responsible for inflammatory diseases (40, 41). Mammalian SIGIRR could inhibit the TLRs signal conduction events by interacting with MyD88 (42–44). The DIGIRR, the homologous of SIGIRR, was only reported in some fish species (27, 28). In the current study, DIGIRR was firstly identified from golden pompano. The trDIGIRR shared high identities with fish DIGIRR and possessed typical structural characteristics of IL-1R family, including extracellular Ig domains, transmembrane region, intracellular conserved TIR domain. Similar to DIGIRR of miiuy croaker and Chinese sturgeon, trDIGIRR also contained two Ig domains (**Figures 1A**, **2B**). Only One Ig domain was found in SIGIRR of mammals, grass carp and zebrafish (**Figures 2A, B**). The Ig domain of IL-1R family members is crucial for ligands recognition (45). Further structural comparisons revealed that the structures of TIR domain of fish DIGIRR or SIGIRR were similar, indicating both DIGIRR or SIGIRR might possessed similar signaling transduction. Although the structure of Ig domain in DIGIRR and SIGIRR was also similar, however the number of Ig domain varied in DIGIRR and SIGIRR, suggesting that DIGIRR might have different mechanism for ligands recognition from that of SIGIRR. We found that trDIGIRR had a signal peptide at its N-terminal, similar to DIGIRR of miiuy croaker, Chinese sturgeon and large yellow croaker, while mammalian SIGIRRs did not had signal peptide (**Figure 1A**), suggesting that fish DIGIRR might be a secretory protein (28). Sequence alignment analysis revealed that the TIR domains of vertebrates’SIGIRR or DIGIRR were highly conserved, all of which contained three Box motifs (Box1-3) (**Figure 2A**). Box1 and Box2 involved in the downstream signal transduction, and Box3 play important roles in cell localization (46). These results suggested that DIGIRR and SIGIRR might share similar downstream signal transduction, which was also confirmed by structural analysis (**Figure 2B**). In addition, we found the two amino acids sites, important for signal transduction, in SIGRR were cystines and Ala-Leu, and these of DIGIRR were replaced by Ser and Ala-Leu, and these of IL-1R were Ser and Arg-Tyr (**Figure 2C**). It had been hypothesized that DIGIRR was a “transitional molecule” from IL-1R to SIGIRR (27, 28). Further analysis revealed that trDIGIRR protein was mainly distributed in the cytoplasm of HET-293T cells (**Figure 4B**), which was line with that of miiuy croaker DIGIRR in Hela cells and pufferfish DIGIRR in HEK-293T, HepG2 and Hela cells (27, 28), suggesting that fish DIGIRR might function in the cytoplasm. However, SIGIRR of mammals and zebrafish were mainly located on the cell membrane. These results indicated that DIGIRR and SIGIRR might play roles in different parts of cells (26, 47).

Fish DIGRRRs were ubiquitously expressed in all tested tissues (27, 48). Similar results were also observed for that of trDIGIRR (**Figure 4A**). The expression of trDIGIRR was highly expressed in intestine, followed by liver, and lowly expressed in heart. The expression patterns of trDIGIRR were in agreement with that of Chinese sturgeon DIGIRR (29), indicating that fish DIGIRR might involve in various life processes. Distinct expressions of DIGIRR were observed. The DIGIRR of miiuy croaker DIGIRR was highly expressed in liver and lowly in intestine, gill and heart (49), and that of puffer fish DIGIRR was mostly expressed in gill (28). Also, fish DIGIRR had different expression patterns from that of fish SIGIRR. Zebrafish SIGIRR was highly expressed in HK and liver, and lowly in heart (26), grass carp SIGIRR was highly expressed in liver, but lowly in brain. In contrast, mammalian SIGIRRs were highly expressed in HK and intestine (18, 30, 50). These results implied that the expressions of DIGIRR or SIGIRR in different species might be species-specific and tissue-specific. The intestine, skin and gill are the main mucosal immune organs of fish (51). HK and spleen are the main immune organs of fish, which contain many immune cells, such as lymphocytes, macrophages and granulocytes (52). High expressions of trDIGIRR in intestine and HK were in line with that of DIGIRR of Chinese sturgeon, grass carp and SIGIRR of zebrafish (26, 30, 52), indicating the important role of DIGIRR or SIGIRR in the immune response of teleost.

Following LPS challenge, the expression of trDIGIRR was down-regulated at early time of challenge and then was significantly up-regulated in various tissues, indicating that trDIGIRR involved in the LPS-induced immune response. The later induced expression by LPS might take parts in the preventing overreaction of inflammation, as a large number of pro-inflammatory were induced by LPS in fish (53, 54). It had been found that pufferfish DIGIRR inhibited the LPS-induced activation of NF-κB pathway in embryonic cells (28). The expression of grass carp SIGIRR was significantly down-regulated in primary splenic cells by LPS stimulation (30). The expression of mice SIGIRR was significantly down-regulated form 6 h to 12 h, and then returned to normal level at 24 h following LPS stimulation (18). These results demonstrated that DIGIRR or SIGIRR play similar roles in the LPS-induced inflammatory response. Follow poly I:C stimulation, the expression of trDIGIRR was significantly increased in liver, intestine, HK and spleen, which was in line with that of grass carp SIGIRR, indicating that DIGIRR or SIGIRR also involved in the polyI:C induced immune response. Post *V. alginolyticus* infection, the expression of trDIGIRR was significantly down-regulated at 6 h in spleen, HK and intestine, and then increased as infection time extension. Similar results were also observed for DIGIRR of Chinese sturgeons after *Mycobacterium marinum* challenge and SIGIRR of grass carp after *Flavobacterium cloumnare* challenge (29, 30), suggesting that DIGIRR or SIGIRR had similar functions during bacterial infection.

Mammalian SIGIRR could negatively regulate the TLRs signaling pathway, by competitively interacting with the adaptor molecules (such as MyD88, IRAKs and TRAF6) through its TIR domain to weaken the excessive TLRs response (55–57). Whilst, zebrafish SIGIRR could not only interact with MyD88 in HEK-293T cells, but also interact with TRIF protein *in vitro* and zebrafish embryo cells, playing important roles in inhibiting the liver inflammation of zebrafish (26). We found that trDIGIRR could only interact with trMyD88, but not interact with trTRIF in HET-293T cells, which was different from that of zebrafish SIGIRR. Our results indicated that trDIGIRR might involve in the inhibition of excessive inflammation though the myD88-dependent signaling pathway. The mechanism needs to be further studied.

In conclusion, DIGIRR was identified in golden pompano and its sequence features were analyzed. The DIGIRR of golden pompano shared high sequence identities with other fish DIGIRR and SIGIRR. Further studies revealed that the expressions of trDIGIRR could be affected by *V. alginolyticus*, LPS and poly I:C challenge, indicating its roles in responses to pathogen invasion. We also demonstrated that trDIGIRR was mainly located in the cytoplasm and could interact with MyD88, but not interact with TRIF. To the best of our knowledge, this is the first reports on DIGIRR of golden pompano. Our results contribute to further understanding the immune roles of fish DIGIRR.

## Data availability statement

The datasets presented in this study can be found in online repositories. The names of the repository/repositories and accession number(s) can be found below: https://www.ncbi.nlm.nih.gov/genbank/, MW239681.

## Author contributions

ZQ and YW designed the experiments, wrote the paper. YX and SG conducted qPCR and co-IP. YX done the structural construction. YJ and QZ done the sequence analysis. All authors contributed to the article and approved the submitted version.

## Funding

This study was supported by the National Natural Science Foundation of China (Grant no. 31860736) and partially by the Major Projects of Natural Science Research for University and Colleges in Jiangsu Province (Grant No. 21KJA240001) and partial by the Projects for the Key Research and Development Program of Jiangsu Province (Grant No. BE2018348) and High-quality Development of Fishery Industry of Yancheng City (Grant No. YCSCYJ20210014). ZQ was supported financially by the projects for “six talents” of Jiangsu Province (Grant No. NY-115).

## Conflict of interest

The authors declare that the research was conducted in the absence of any commercial or financial relationships that could be construed as a potential conflict of interest.

## Publisher’s note

All claims expressed in this article are solely those of the authors and do not necessarily represent those of their affiliated organizations, or those of the publisher, the editors and the reviewers. Any product that may be evaluated in this article, or claim that may be made by its manufacturer, is not guaranteed or endorsed by the publisher.
